# Relationship between STarT Back Screening Tool and prognosis for low back pain patients receiving spinal manipulative therapy

**DOI:** 10.1186/2045-709X-20-17

**Published:** 2012-06-12

**Authors:** Jonathan Field, Dave Newell

**Affiliations:** 1 Private practice Back2Health, 2 Charles Street, Petersfield, Hants, UK; 2 Anglo-European College of Chiropractic, 13-15 Parkwood Rd, Bournemouth, UK

**Keywords:** Start back tool, Low back pain, Prognosis, Spinal manipulative therapy

## Abstract

**Background:**

Low back pain (LBP) is common and costly and few treatments have been shown to be markedly superior to any other. Effort has been focused on stratifying patients to better target treatment. Recently the STarT Back Screening Tool (SBT) has been developed for use in primary care to enable sub grouping of patients based on modifiable baseline characteristics and has been shown to be associated with differential outcomes. In the UK the SBT is being recommended to assist in care decisions for those presenting to general practitioners with LBP. In the light of growing recommendation for widespread use of this tool, generalisability to other LBP populations is important. However, studies to date have focused only on patients attending physiotherapy whereas LBP patients seeking other treatment have not been investigated.

**Aims:**

This study aims to investigate the utility of the SBT to predict outcomes in LBP patients presenting for chiropractic management.

**Methods:**

A total of 404 patients undergoing chiropractic care were asked to complete the SBT before initial treatment. Clinical outcomes were collected at 14, 30 and 90 days following this initial consultation. The clinical course was described comparing SBT categories and logistic regression analysis performed to examine the tool’s prognostic utility.

**Results:**

Although the high-risk categories had greater pain at baseline this difference rapidly faded, with both change in composite outcome scores and pain scores being statistically insignificant between the risk groups at 30 and 90 days follow up. In addition, both univariate and adjusted analysis showed no prognostic utility of the SBT categorisations to differentiate clinical outcomes between risk groups.

**Conclusion:**

Whilst the SBT appears useful in some back pain populations it does not appear to differentiate outcomes in LBP patients seeking chiropractic care.

## Introduction

Low back pain (LBP) is a common symptom causing health-seeking behavior in up to half of those who experience it [1,2]. Between six and nine percent of the UK population consult their general practitioner (GP) for LBP each year, accounting for 5 million GP consultations annually [3,4]. For most of these patients a low back pain episode will most likely be a temporary inconvenience, yet for a minority who have sought care (approximately 28%) it becomes an enduring and disabling problem [5,6].

The extent of the costs to society of this syndrome have led to the call for identification of potential subgroups of non specific low back pain (nsLBP) in the belief that this group consists of a heterogeneous mix of presentations and etiologies. Identification of groups of back pain that respond better with specific interventions would facilitate targeted treatment [7]. In addition, evidence-based guidelines highlight the need to consider prognostic factors when deciding the management of nsLBP [8-10], where early identification of potential barriers to recovery may help guide treatment aimed at secondary prevention of persistent back pain [11,12].

In the absence of serious pathology, recovery from back pain in individuals from the general back pain population as well as those seeking help from chiropractors is only weakly related to physical findings [13-17], with only a small number of condition specific factors associated with poor prognosis [13,18,19]. However, psychological factors are found to influence future disability, pain and self reported improvement in LBP patients presenting to GPs, secondary care services and surgery [20-23]. This has led to guidelines recommending that non-physical factors be considered when setting the treatment for LBP patients [8-10]. In chiropractic LBP populations the significance of psychological factors is less certain as exploratory studies have found little or no correlation with outcomes [14,24-26].

The STarT Back Tool (SBT) has recently been developed to help primary care practitioners make care decisions about the likely need LBP patients have for secondary prevention based on modifiable risk factors for poor outcome [20]. The SBT places patients into one of three categories (Low, Medium and High) of risk for having persisting LBP with disability. In a recent trial, patients whose care had been stratified using the SBT to receive either advice alone, ‘standardised’ physiotherapy or psychologically based care with physiotherapy had lower disability at 4 and 12 months than those patients undergoing usual care as directed by the clinical judgment of a physiotherapist [27]. As a consequence of those studies the SBT is being recommended by commissioning services in the UK NHS to guide care pathways for those presenting to GP with LBP. Whilst the feasibility of using the SBT in a chiropractic patient population has been demonstrated [28], as yet, no appraisal of the prognostic utility of grouping individuals seeking chiropractic care has been published.

This paper investigated whether nsLBP patients classified in the high-risk (complex psychosocial) group by the SBT do less well with chiropractic care than those either at low risk or medium risk groups.

## Methods

### Subjects and procedure

Consecutive patients aged over 16 presenting with nsLBP to one of six chiropractic clinics in the south of England were asked, as part of normal practice to complete the Bournemouth Questionnaire (BQ) [29] either at the clinic or on-line before their first visit. Those doing so on line were additionally asked to complete the SBT. Patients were presented with a consent form when they completed pre-examination forms online, via a web page. In these practices patients who start treatment are emailed outcome assessment questionnaires consisting of the BQ and a Patients Global Impression of Change (PGIC), at 14, 30 and 90 days following their initial visit.

### Outcomes

For this study, the primary outcome was the PGIC. In addition we also measured pain as derived from the pain sub-scale of the BQ and total BQ scores. Patients were categorised into the three SBT risk groups using the method as described by Hill et al. [20,30]. For each of the follow up points, all outcomes were dichotomized. Thus poor outcome was defined by a PGIC response of better or much better (score of <6) [31], a change in pain of less than or equal to (≤)2 points [32] and a change in total BQ of ≤ 46% [33]. Recently, both the PGIC and BQ have been recommended as preferred measurements by the ‘Any Qualified Provider Resource Centre’ (UK, NHS) for monitoring outcomes in low back pain patients [34].

### Analysis

General characteristics of the patient sample were calculated as means and proportions with appropriate measures of variance. Differences between categorical baseline characteristics were determined using *χ*^2^, or Pearson *χ*^2^ test for trend. Further, a Kolmogorov-Smirnov test revealed significant deviation from Gaussian distributions for pain and total BQ data, despite using logarithmic transformation. Consequently differences within these variables across SBT categories were analysed using Kruskal Wallis tests, whereas change scores revealed a Gaussian distribution and were analysed using a one-way ANCOVA adjusted for baseline scores.

To determine any associations between baseline SBT categorisation and the outcomes univariate logistic regression analysis was carried out using the SBT categorisation as the independent variable and the dichotomised outcomes (PGIC, change in pain and change in total BQ) as dependent variables at each of the follow up time points. This analysis was repeated after sub grouping by gender, but only for the PGIC as a dependent variable. Finally, adjusted models for predicting poor outcome as defined by the PGIC were constructed with an entry criterion for significant variables of p < 0.15 and retention at p < 0.05 using a binary logistic analysis forward LR procedure. All statistical analyses were performed using statistical software SPSS (v17.0, SPSS Inc., Chicago IL).

### Ethics

Ethics for this study were sought and approved by the Research and Ethics subcommittee of the Anglo-European College of Chiropractic Research Committee.

## Results

**Figure 1:**
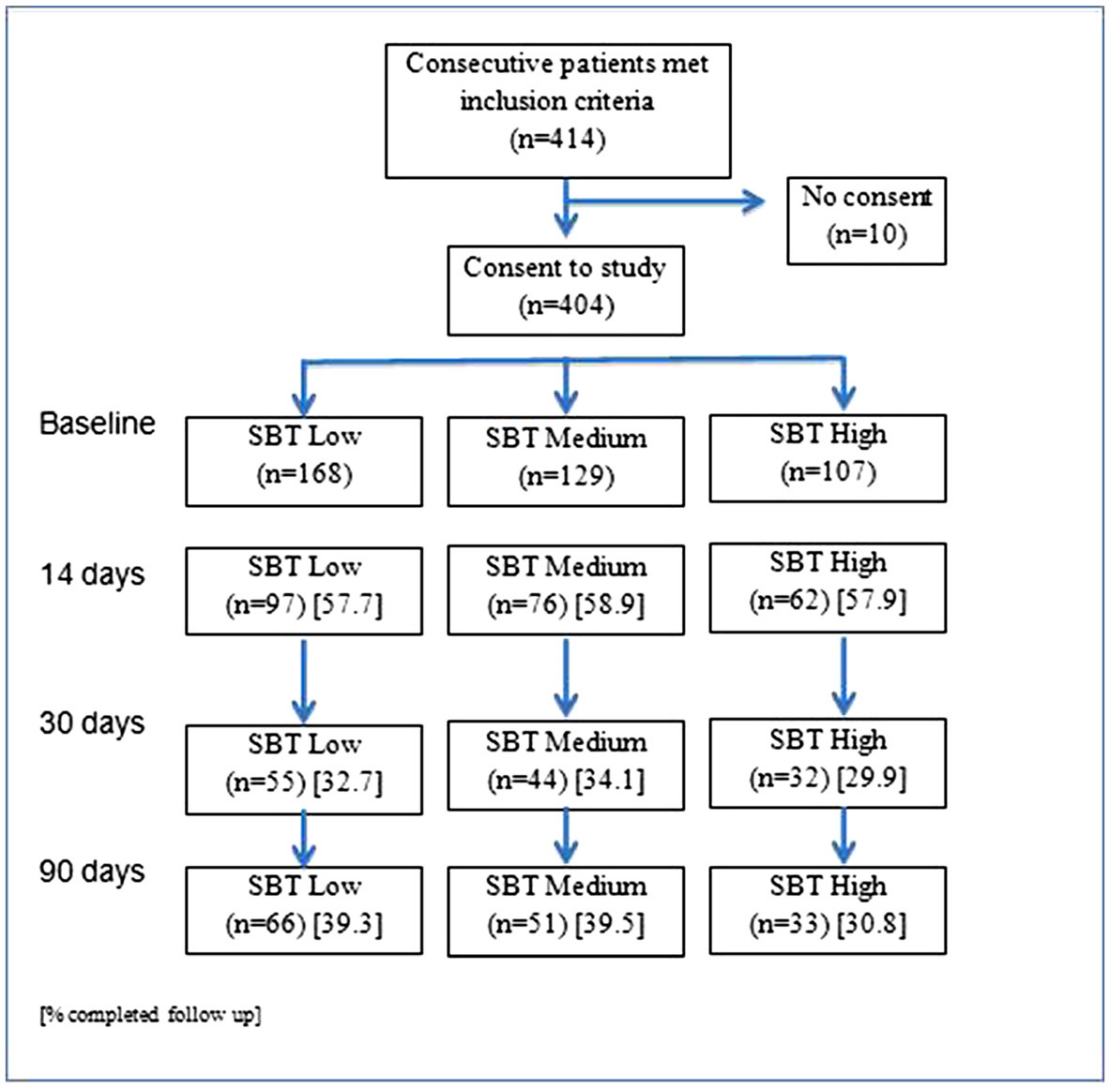
Numbers in study at each follow up point in each Start Back Tool category.

A total of 819 patients presented with nsLBP between March and November 2011. Four hundred and five were ineligible to participate in the study, as they did not complete the initial forms online. There was no difference (p > 0.05) in gender, duration, or BQ scores at presentation or in pain and BQ scores at 90 days follow up between those submitting the forms online and those doing so at the clinic. However at presentation those completing the forms online and so being included in this study had slightly less pain (mean (SD) 6.2(2.0) versus 6.6(2.2), p0.04) and were younger (44.7(15) versus 48.9(15.4) years, p <0.01). Four hundred and four individuals completing the forms online were categorized by the SBT at the study inception. Of these 168 (41.6%), 129 (31.9%) and 107 (26.5%) were at low, medium or high risk of poor outcome at baseline respectively. Numbers of participants at follow up within each SBT group and percentage response rates are shown in Figure 1. The number of treatments received across the three SBT categories were; low (4.1(2.4)); medium (4.3 (2.4)) and high (4.4 (2.6)). There were no significant differences in treatment visits between SBT categories (p = 0.54).

Table 1 shows the demographic and condition specific characteristics within each SBT risk group. Only the proportions of those patients with pain above the knee, higher baseline pain and greater total BQ scores differed between the SBT categories. The distribution of duration of back pain in the complete sample was 56.2%, 12.4% and 31.4% for less than 1 month, 1–3 months and greater than 3 months respectively. These proportions were similar across the SBT risk groups.

**Table 1:** Descriptive analysis of baseline variables across Start Back Tool (SBT) categories

**Variables**	**SBT Category**		
	**Low (n = 168)**	**Medium (n = 129)**	**High (n = 107)**
**Mean age (SD)**	45.4 (15.1)	45.9 (15.0)	45.8 (14.1)
**Female**	54.8%	52.7%	50.5%
**Seen practitioner before**	47.0%	59.7%	33.6%
**Leg Pain**			
Above the knee^*^	25.5%	23.3%	35.5%
** Below the knee**	6.0%	10.9%	12.1%
**>30 days pain in year**	41.1%	41.1%	38.3%
**Recurring**	69.6%	65.9%	59.8%
**Duration**			
** < 1 month**	54.8%	53.5%	61.7%
** 1**–**3 months**	14.3%	14.0%	7.5%
** >3 months**	31.0%	32.6%	30.8%
Median Pain (25, 75)^**^	5 (4–7)	7 (6–8)	7 (6–9)
Median BQ (25, 75)^**^	24 (14–33)	36 (30–44)	45 (34–54)

*P < 0.05 (Chi^2^ test for trend). **p < 0.001, (Kruskal-Wallis).

**Figure 2:**
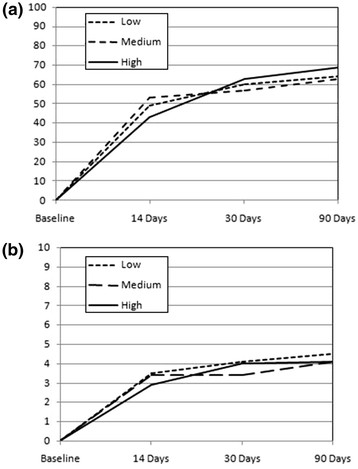
Resdualised^*^change scores compared between SBT risk groups for Total BQ (a) and Pain (b).

Table 2 shows the total BQ and pain scores over time for each of the SBT categories. Pain and total BQ scores differed significantly between the SBT risks groups at baseline, where high-risk groups started with higher scores (p < 0.001). The total BQ scores also differed at 14 days follow up (p < 0.001). However, any differences between the risk groups were absent by 30 days with all groups achieving very similar scores by 90 days. Mean residual change scores for total BQ and pain are illustrated in Figure 2. These changes are not significantly different between risk categories at any of the follow up time points (Change in Pain: 14 days (ANCOVA. F = 1.1, p = 0.32); 30 days (ANCOVA. F = 1.4, p = 0.25); 90 days (One way ANCOVA. F = 0.5, p = 0.62) Change in total BQ: 14 days (ANCOVA. F = 0.8, p = 0.46); 30 days (ANCOVA. F = 0.2, p = 0.83); 90 days (ANCOVA. F = 0.2, p = 0.83)).

**Table 2:** Pain, total BQ scores and proportion of subjects with a poor outcome across Start Back Tool (SBT) categories at 14, 30 and 90 days follow up

**SBT Category**	**14 days (n = 235)**		**30 days (n = 131)**		**90 days (n = 150)**	
	**Median Pain (25, 75)**	Median BQ (25, 75)^**^	**Median Pain (25, 75)**	**Median BQ (25, 75)**	**Median Pain (25, 75)**	**Median BQ (25, 75)**
**Low**	2 (1–3)	10 (3–18)	1 (1–3)	6 (2–14)	1 (1–2)	5 (0–15)
**Medium**	2 (1–5)	12 (3–26)	2 (1–4)	10 (5–21)	1 (1–4)	8 (0–18)
**High**	3 (1–5)	20 (8–33)	2 (0–3)	6 (2–20)	2 (0–3)	10 (3–22)

**p < 0.001, (Kruskal-Wallis), ^¥^Patients Global Impression of Change < 6.

Table 3 shows the proportion of patients with poor outcome in each SBT group at each follow up point as defined by three dichotomized outcomes. A univariate logistic regression analysis revealed that SBT categorisations were not statistically associated with the primary outcome (PGIC) at any of the follow up points (Table 4) However, the utility of the SBT to discriminate poor outcome using the PGIC did increase over time, with both medium and high risk groups being around 1.7 times the odds of poor outcome by 90 days compared to low risk groups, albeit not statistically significant. A further analysis using this outcome but sub grouping by gender revealed a similar, but again statistically insignificant association. In this analysis only male patients showed any marked association between baseline categorisation by SBT and poor outcome, where those in the medium and high risk groups had 3 times the odds of experiencing poor outcome compared to low risk groups at 90 days. In females however, the SBT had little predictive utility at any follow up point. (Table 5).

**Table 3:** Proportions (%) of patients with a poor outcome across Start Back Tool categories defined by cut-off points for each outcome

**Outcomes**	**14 days (n)**	**30 days (n)**	**90 days (n)**
**PGIC**			
Low	36.1 (35)	29.1 (16)	18.2 (12)
Medium	30.3 (23)	29.5 (13)	27.5 (14)
High	33.9 (21)	28.1 (9)	27.3 (9)
**≤2 on NRS**			
Low	37.9 (33)	24.0 (12)	23.0 (14)
Medium	24.7 (18)	26.2 (11)	24.5 (12)
High	32.8 (19)	32.3 (10)	18.8 (6)
**≤47% on BQ**			
Low	28.9 (24)	20.4 (10)	14.0 (8)
Medium	29.4 (20)	25.6 (10)	19.6(9)
High	41.5 (22)	29.0 (9)	18.2 (6)

*PGIC* = Patient Global Impression of Change; *NRS* = Numerical Rating Scale; *BQ* = Bournemouth Questionnaire.

**Table 4:** Predicting poor outcome (PGIC) at 14, 30 and 90 days for the whole group (n = 404)

**SBT Category**	**14 days (n = 235)**	**30 days (n = 131)**	**90 days (n = 150)**
	**OR (95% CI)**	**OR (95% CI)**	**OR (95% CI)**
**Low**	1.0	1.0	1.0
**Medium**	0.8 (0.4 to 1.5)	1.0 (0.4 to 2.4)	1.7 (0.7 to 4.1)
**High**	0.9 (0.5 to 1.8)	0.9 (0.4 to 2.5)	1.7 (0.6 to 4.5)

*PGIC* = Patient Global Impression of Change; *SBT* = Start Back Tool.

**Table 5:** Predicting poor outcome (PGIC) at 14, 30 and 90 days follow up split by gender

**SBT Category**	**14 days**		**30 days**		**90 days**	
	**OR (95% CI)**		**OR (95% CI)**		**OR (95% CI)**	
	**Male (n = 117)**	**Female (n = 118)**	**Male (n = 66)**	**Female (n = 65)**	**Male (n = 71)**	**Female (n = 79)**
**Low**	1.0	1.0	1.0	1.0	1.0	1.0
**Medium**	1.2 (0.5 to 3.0)	0.5 (0.2 to 1.3)	0.7 (0.2 to 2.7)	1.2 (0.3 to 4.0)	3.0 (0.9 to 10.0)	0.9 (0.2 to 3.3)
**High**	1.8 (0.7 to 5.0)	0.5 (0.2 to 1.2)	0.7 (0.2 to 3.2)	1.0 (0.3 to 4.1)	3.0 (0.6 to 16.0)	1.1 (0.3 to 4.0)

*PGIC* = Patient Global Impression of Change; *SBT* = Start Back Tool.

Categorising poor outcome as ≤2 points drop in pain, a further analysis revealed no significant association between the SBT and an absence of meaningful change in pain at any follow up point (Table 6). This result was also apparent when using dichotomized total BQ change scores as the dependent variable (Table 7).

**Table 6:** Predicting poor outcome (Change in pain ≤ 2 points) at 14, 30 and 90 days

**SBT Category**	**14 days (n = 218)**	**30 days (n = 123)**	**90 days (n = 142)**
	**OR (95% CI)**	**OR (95% CI)**	**OR (95% CI)**
**Low**	1.0	1.0	1.0
**Medium**	0.5 (0.3 to 1.1)	1.1 (0.4 to 2.9)	1.1 (0.4 to 2.6)
**High**	0.8(0.4 to 1.6)	1.5 (0.5 to 4.1)	0.8 (0.3 to 2.2)

*SBT* = Start Back Tool.

**Table 7:** Predicting poor outcome (Change in total BQ ≤47%) at 14, 30 and 90

**SBT Category**	**14 days (n = 204)**	**30 days (n = 119)**	**90 days (n = 136)**
	**OR (95% CI)**	**OR (95% CI)**	**OR (95% CI)**
**Low**	1.0	1.0	1.0
**Medium**	1.0 (0.5 to 2.1)	1.3 (0.5 to 3.6)	1.5 (0.5 to 4.2)
**High**	1.7 (0.8 to 3.6)	1.6 (0.6 to 4.5)	1.4 (0.4 to 4.3)

*BQ* = Bournemouth Questionnaire; *SBT* = Start Back Tool.

A forward LR logistic analysis adjusting for all potential baseline predictors (Enter: p ≤ 0.15, Retain p ≤ 0.05) resulted in few variables remaining as independent predictors of outcome at the follow up points, with baseline SBT categorisations failing to be retained in any of the models (Table 8). Only duration, reoccurrence of the problem and pain for greater than 30 days in the last year provided any prognostic utility and even here the degree of variance explained remained relatively low as did the predictive accuracy.

**Table 8:** Adjusted models for predicting poor outcome (PGIC) at 14, 30 and 90 days follow up

**Follow up point**	**Variables in the equation**	**OR (95% CI)**	Nagelkerke^*^	**sn/sp**
**14 days (n = 235)**	Pain for >30 days in year	3.2 (1.9 to 5.6)	0.26	90/25
	Pain (for every 1 point increase)	0.8 (0.8 to 0.9)		
**30 days (n = 131)**	Duration		0.32	92/29
	< 1 month	1.0		
	1–3 months	5.6 (1.8 to 17.0)		
	>3 months	2.4(1.1 to 5.5)		
**90 days (n = 150)**			0.45	94/23
	Duration			
	< 1 month	1.0		
	1–3 months	9.3 (3.0 to 29.0)		
	>3 months	3.0(1.3 to 7.0)		

* = variance explained by model; *sn* = sensitivity; *sp* = specificity; *PGIC* = Patient Global Impression of Change.

## Discussion

In this study those categorised by the SBT as being at low risk of having a poor prognosis were somewhat more likely to do well compared to those placed in the medium or high risk groups. However this difference was small and failed to reach any statistical significance in a univariate analysis. In addition the adjusted models at each follow up did not include the SBT tool as a significant predictor of outcome. This result suggests that, in this population at least, the proportion of high-risk patients that improve is not significantly different from those at medium or low risk.

Patients placed in the SBT high-risk group had more adverse BQ and pain scores at presentation. However, there was a greater improvement in these scores in the high-risk group with the result that by the 30-day assessment this difference was no longer evident. These results are partially corroborated by Fritz et al. [35] who showed that despite high risk patients starting with more pain and disability at baseline they experienced greater improvement over a course of care.

The SBT has been developed to better stratify LBP patients for targeted treatment. Hill et al. (2008) [20] showed the discriminative ability of this tool to differentiate those who clinically changed on the RMDQ. This was largely based on differences in barriers to recovery, with the high-risk group being defined as having psychological barriers and the medium as having physical barriers. These authors went on to show that the SBT was able to support treatment choice more efficiently than physiotherapists’ clinical experience alone. Although this approach has potential to improve outcomes and cost in LBP the generalisability of their results may not be supported by this study.

There may be a number of reasons why this is so. Firstly, this LBP population differs from previous studies in that they were largely self-selecting and sought chiropractic care privately. In addition to this, around 41% of this sample had experienced chiropractic care from the same practitioner previously. This may have led to higher expectations of success which may have impacted on the psychological response of people in this sample. Another possibility is that patients in the various SBT categories within our cohort study received different types of care and that this influenced their recovery. However, communicating with the participating clinicians indicated that they had not accessed the SBT category data, and there were no differences in the number of treatments provided between SBT categories. Despite this, differences in treatment approach cannot be ruled out. It is possible that those placed in the high risk SBT category received care that was consciously or unconsciously delivered by the chiropractor in a way so as to address the patient’s psychological requirements. As a development of this idea, the treatment interventions in this study may inherently be similar to the treatment intervention given in Hill et al. (20) for their SBT high-risk group. This would mean that all groups in this study may have received the treatment reserved in the Hill et al. study (20) exclusively for the high risk group, thus masking any initial prognostic information given by the SBT at baseline. However, any treatment effects must be viewed as strongly speculative due to this study’s purely observational design.

It has been suggested that patients presenting to chiropractors are a psychologically healthy subgroup with few individuals having levels of adverse psychological factors sufficient to influence their prognosis [24]. In support of this, a narrative review comparing reports of psychological questionnaires applied to chiropractic populations and non-chiropractic populations where the same test tools have been used [36], concluded that those seeing chiropractors were less likely to have adverse scores across a range of psychological domains that have previously been linked to poor prognosis in other LBP populations. In this study 26.5% of patients were categorised by the SBT as in the high-risk group. This suggests that either a significant proportion had potentially adverse psychology, or that the SBT is inappropriately categorizing some of those in this population.

The failure of SBT categorisation in this LBP population to identify those less likely to improve may be because, as a self-selecting subgroup, these individual possess features that negate the impact of otherwise adverse psychology. The possible existence of ‘protective’ factors in some populations is supported by the presence of higher pain related self-efficacy reducing the impact of raised fear avoidance beliefs in patients with chronic LBP [37]. Reports from one chiropractic study that has looked at this found more favorable levels of self-efficacy than other reported populations, and higher levels of self-efficacy were found to relate to a better prognosis [26,36].

Alternatively a reduction in levels of adverse psychological variables may have occurred during care, therefore reducing their effect on treatment response. In support of this, a systematic review of psychological outcomes from studies involving manipulation describes significantly greater reduction in adverse scores on psychological questionnaires in populations having manipulation when compared to groups receiving verbal interventions (advice, education or handout) or other physical treatments (exercise, electrotherapy, sham manipulation or acupuncture) [38]. In addition to this, a small study found that a statistically significant reduction in fear avoidance beliefs and catastrophisation as well as improvement in self-efficacy occurred shortly after an initial visit with a chiropractor. Despite baseline levels of these variables not relating to self-reported outcome at one month, post-visit scores did display a weak but significant relationship to outcomes, with those retaining two or more higher variables post-visit having increased odds of a poor outcome [26].

Caution however, must be used when interpreting these findings. Firstly, this was not a clinical trial investigating treatment effects and as such it is not possible to ascertain any treatment impact on changes in patients’ psychology or symptoms. In addition this population of patients had been drawn from a group of six linked practices and as such may not be representative of the wider population seeking privately funded treatment for LBP.

Additionally, there are some differences in response rates between the SBT categories, particularly at 90 days and the potential remains for selection bias through differential attrition. However, comparisons of baseline demographics (age, pain, duration and BQ score) for each of the three SBT categories demonstrated no statistically significant differences between responders and non responders, as was the number of treatments received at 90 days. Although this cannot rule out the possibility that those responding experienced different outcomes to non responders it provides some support for the contention that responders were more likely to be a representative sample based upon care received and baseline factors.

Lastly, comparisons of the results here with other studies are problematic due to differences in follow up times, outcome instruments and other methodological differences.

## Conclusion

This study has shown that LBP patients seeking treatment for chiropractic and categorised by SBT at baseline show no differential risk of poor outcome between categorisation groups. Although this tool does differentiate LBP patients in terms of baseline pain and baseline total BQ scores, these differences disappeared by 30 days. At the present time, it is unclear whether the SBT is transferable to LBP populations outside of those it was originally developed for.

## Competing interests

The author(s) declare that they have no competing interests.

## Authors’ contribution

JF conceived of the study, and was involved with its design, data collection, statistical analysis, interpretation and drafting the manuscript. DN performed the statistical analysis and was involved with its interpretation and in drafting the manuscript. Both authors read and approved the final manuscript.

## References

[B1] Hillman M Wright A Rajaratnam G Tennant A Chamberlain MA Prevalence of low back pain in the community: implications for service provision in Bradford, UK J Epidemiol Community Health 1996 50 3 347 352 10.1136/jech.50.3.347 8935469 PMC1060294

[B2] Waxman R Tennant A Helliwell P Community survey of factors associated with consultation for low back pain BMJ 1998 317 7172 1564 15647 10.1136/bmj.317.7172.1564 9836660 PMC28737

[B3] Croft PR Macfarlane GJ Papageorgiou AC Thomas E Silman AJ Outcome of low back pain in general practice: a prospective study BMJ 1998 316 7141 1356 1359 10.1136/bmj.316.7141.1356 9563990 PMC28536

[B4] Dunn K Classification of low back pain in primary care: using “bothersomeness” to identify the most severe cases Spine 2005 30 16 1887 1892 10.1097/01.brs.0000173900.46863.02 16103861

[B5] Hestbaek L Leboeuf-Yde C Manniche C Low back pain: what is the long-term course? A review of studies of general patient populations Eur Spine J 2003 12 2 149 165 12709853 10.1007/s00586-002-0508-5 PMC3784852

[B6] Henschke N Maher CG Refshauge KM Herbert RD Cumming RG Bleasel J Prognosis in patients with recent onset low back pain in Australian primary care: inception cohort study BMJ 2008 337 171 10.1136/bmj.a171 PMC2483884 18614473

[B7] Bouter LM Pennick V Bombardier C Cochrane back review group Spine 2003 28 12 1215 12811262 10.1097/01.BRS.0000065493.26069.1C

[B8] vanTulder M Becker A Bekkering T Breen A Gil del Real M Hutchinson A European guidelines for the management of acute nonspecific low back pain in primary care Eur Spine J 2006 15 169 15 16 10.1007/s00586-006-1071-2 PMC3454540 16550447

[B9] Chou R Quseem A Snow V Casey D Cross T Shekelle P Diagnosis and Treatment of Low Back Pain: A Joint Clinical Practice Guideline from the American College of Physicians and the American Pain Society Ann Intern Med 2007 2 147 478 147 10.7326/0003-4819-147-7-200710020-00006 17909209

[B10] Savigny P Watson P Underwood MR NICE Guideline Development Group NICE Guideline Development Group Early management of persistent non-specific low back pain: summary of NICE guidance BMJ 2009 338 10.1136/bmj.b1805 19502217

[B11] Jellema P Van Der Windt D Prediction of an unfavourable course of low back pain in general practice: comparison of four instruments Br J General Practice 2007 57 534 15 22 PMC2032695 17244419

[B12] Melloh M Elfering A EgliPresland C Roeder C Barz T RolliSalathh C Identification of prognostic factors for chronicity in patients with low back pain: a review of screening instruments International Orthopaedics (SICOT) 2009 33 2 301 301 10.1007/s00264-008-0707-8 PMC2899092 19130056

[B13] Leboeuf-Yde C Greboeuf-Y A Borge JA Lothe J Magnesen E Nilsson Nilssoal The nordic back pain subpopulation program: demographic and clinical predictors for outcome in patients receiving chiropractic treatment for persistent low back pain JMPT 2004 27 8 493 502 15510092 10.1016/j.jmpt.2004.08.001

[B14] Newell D Field J Who will get better? Predicting clinical outcomes in a chiropractic practice Clin Chiropr 2008 13 1 108

[B15] Lakke S Soer R Takken T Reneman M Risk and prognostic factors for non-specific musculoskeletal pain: A synthesis of evidence from systematic reviews classified into ICF dimensions Pain 2009 147 3 153 164 10.1016/j.pain.2009.08.032 19800735

[B16] Chou R Shekelle P Will this patient develop persistent disabling low back pain? JAMA 2010 303 13 1295 1302 10.1001/jama.2010.344 20371789

[B17] Ramond A Bouton C Richard I Roquelaure Y Baufreton C Legrand E Psychosocial risk factors for chronic low back pain in primary care--a systematic review Fam Pract 2011 28 1 12 21 10.1093/fampra/cmq072 20833704

[B18] Thomas E Silman AJ Croft PR Papageorgiou AC Jayson MIV Macfarlane GJ Predicting who develops chronic low back pain in primary care: a prospective study BMJ 1999 318 1662 1667 10.1136/bmj.318.7199.1662 10373170 PMC28145

[B19] Bolton JE Hurst H Prognostic factors for short-term improvement in acute and persistent musculoskeletal pain consulters in primary care Chiropr Man Therap 2011 19 27 10.1186/2045-709X-19-27 PMC3269996 22078488

[B20] Hill JC Dunn KM Lewis M Mullis R Main CJ Foster NE A primary care back pain screening tool: identifying patient subgroups for initial treatment Arthritis Rheum 2008 59 5 632 641 10.1002/art.23563 18438893

[B21] Celestin J Edwards RR Jamison RN Pretreatment Psychosocial Variables as Predictors of Outcomes Following Lumbar Surgery and Spinal Cord Stimulation: A Systematic Review and Literature Synthesis Pain Medicine 2009 10 4 639 653 10.1111/j.1526-4637.2009.00632.x 19638142

[B22] Kamper SJ Maher CG Hancock MJ Koes BW Croft PR Hay EM Treatment-based subgroups of low back pain: a guide to appraisal of research studies and a summary of current evidence Best Practice & Research Clinical Rheumatology 2010 24 2 181 191 10.1016/j.berh.2009.11.003 20227640

[B23] Grotle M Foster NE Dunn KM Croft P Are prognostic indicators for poor outcome different for acute and chronic low back pain consulters in primary care? Pain 2010 151 3 790 797 10.1016/j.pain.2010.09.014 20932646 PMC3398128

[B24] Langworthy J Breen A Psychosocial factors and their predictive value in chiropractic patients with low back pain: a prospective inception cohort study ChiroprOsteopat 2007 15 5 10.1186/1746-1340-15-5 PMC1852566 17394652

[B25] Leboeuf-Yde C Rosenbaum A Axen I Lövgren P Jørgensen K Halasz L The Nordic Subpopulation Research Programme: prediction of treatment outcome in patients with low back pain treated by chiropractors - does the psychological profile matter? ChiroprOsteopat 2009 17 14 10.1186/1746-1340-17-14 PMC2807423 20042095

[B26] Field J Newell D McCarthy PW Preliminary study into the components of the fear-avoidance model of LBP: change after an initial chiropractic visit and influence on outcome ChiroprOsteopat 2010 18 21 10.1186/1746-1340-18-21 PMC2923166 20673330

[B27] Hill JC Whitehurst DG Lewis M Bryan S Dunn KM Foster NE Comparison of stratified primary care management for low back pain with current best practice (STarT Back): a randomised controlled trial Lancet 2011 378 9602 1560 1571 21963002 10.1016/S0140-6736(11)60937-9 PMC3208163

[B28] Kongsted A Johannesen E Leboeuf-Yde C Feasibility of the STarT Back Screening Tool in chiropractic clinics: A cross-sectional study of patients with low back pain Chiropr Man Therap 2011 19 10 10.1186/2045-709X-19-10 PMC3105955 21526986

[B29] Bolton JE Breen A The Bournemouth Questionnaire: a short-form comprehensive outcome measure. I. Psychometric properties in back pain patients JMPT 1999 22 8 503 510 10543579 10.1016/s0161-4754(99)70001-1

[B30] Hill JC Vohora K Dunn KM Main CJ Hay EM Comparing the STarT back screening tool's subgroup allocation of individual patients with that of independent clinical experts The Clinical journal of pain 2010 26 9 783 787 10.1097/AJP.0b013e3181f18aac 20842014

[B31] Newell D Bolton JE Responsiveness of the Bournemouth Questionnaire in determining minimal clinically important change in subgroups of low back pain patients Spine 2010 35 19 1801 1806 10.1097/BRS.0b013e3181cc006b 20581759

[B32] Ostelo RW Deyo RA Stratford P Waddell G Croft P Von Korff M Bouter LM de Vet HC Interpreting change scores for pain and functional status in low back pain: towards international consensus regarding minimal important change Spine 2008 33 1 90 94 10.1097/BRS.0b013e31815e3a10 18165753

[B33] Hurst H Bolton J Assessing the clinical significance of change scores recorded on subjective outcome measures J Manipulative PhysiolTher 2004 27 1 26 35 10.1016/j.jmpt.2003.11.003 14739871

[B34] Extension of Patient choice of Any Qualified Provider in MSK Services for Back and Neck Painhttp://www.supply2health.nhs.uk

[B35] Fritz JM Beneciuk JM George SZ Relationship Between Categorization With the STarT Back Screening Tool and Prognosis for People Receiving Physical Therapy for Low Back Pain Phys Ther 2011 91 5 722 732 10.2522/ptj.20100109 21451094

[B36] Field J Newell D McCarthy P Are chiropractic patients a psychologically self defining subgroup? Clin Chiropr 2011 14 2 72 73

[B37] Woby SR Urmston M Watson PJ Self-efficacy mediates the relation between pain-related fear and outcome in chronic low back pain patients Eur J Pain 2007 11 7 711 718 10.1016/j.ejpain.2006.10.009 17218132

[B38] Williams N Hendry M Lewis R Russell I Westmoreland A Wilkinson C Psychological response in spinal manipulation (PRISM): A systematic review of psychological outcomes in randomised controlled trials Complementary Therapies in Medicine 2007 15 4 271 283 10.1016/j.ctim.2007.01.008 18054729

